# Prediction of GPCR-Ligand Binding Using Machine Learning Algorithms

**DOI:** 10.1155/2018/6565241

**Published:** 2018-01-30

**Authors:** Sangmin Seo, Jonghwan Choi, Soon Kil Ahn, Kil Won Kim, Jaekwang Kim, Jaehyuck Choi, Jinho Kim, Jaegyoon Ahn

**Affiliations:** ^1^Department of Computer Science and Engineering, Incheon National University, Incheon, Republic of Korea; ^2^Department of Life Science, Incheon National University, Incheon, Republic of Korea; ^3^Department of Chemistry, Incheon National University, Incheon, Republic of Korea

## Abstract

We propose a novel method that predicts binding of G-protein coupled receptors (GPCRs) and ligands. The proposed method uses hub and cycle structures of ligands and amino acid motif sequences of GPCRs, rather than the 3D structure of a receptor or similarity of receptors or ligands. The experimental results show that these new features can be effective in predicting GPCR-ligand binding (average area under the curve [AUC] of 0.944), because they are thought to include hidden properties of good ligand-receptor binding. Using the proposed method, we were able to identify novel ligand-GPCR bindings, some of which are supported by several studies.

## 1. Introduction

G-protein coupled receptors (GPCRs) play an important role that involves detecting molecules or ligands from the outside of a cell and activating internal signal transduction pathways and cellular responses. Because the binding of an external ligand and a GPCR induces the coupling of a GPCR and G-proteins, which is followed by various forms of signal transduction, GPCRs have been extensively studied as important drug targets.

An important step for studying GPCRs as a drug target is the identification of drugs or ligands that bind to specific GPCR proteins. Naturally, many biochemistry or bioinformatics approaches to identification of drug-receptor binding have been proposed. Many of these approaches focus on calculating protein-ligand binding affinity [1–3]. However, because those methods rely on 3D structures of proteins or ligands, they cannot be used if the 3D structures of proteins or ligands are not known.

In the other class of protein-ligand identification methods, several features are extracted from proteins and ligands, and machine learning methods, such as SVM (Support Vector Machine) [4–6] or neural network [7], are applied to those features. The key principle of these approaches is that they make use of the similarities between proteins or targets. Jacob and Vert proposed a method that exploits the availability of known ligands for similar targets [6]. Geppert et al. clustered proteases into several groups and used SVM to predict ligands of proteins that have no known binding ligand [4]. Several network-based approaches have also been proposed. Iacucci et al. viewed the issue of protein-ligand binding identification as a protein-protein interaction (PPI) prediction problem [5], while Cheng et al. developed a network-based inference method to identify protein-ligand bindings [8].

In the present study, we propose a novel protein-ligand binding prediction method, which makes use of the local and global structure of a ligand and amino acid motif sequence of a GPCR. The proposed approach is not dependent on the 3D structure of a receptor. Instead, it infers hidden properties of good ligand-receptor bindings, which are encoded as a random forest classifier. We performed extensive testing of various combinations of feature sets of receptor and ligand and found various machine learning algorithms that were effective. The proposed method shows a high average area under the curve (AUC) of 0.944, which indicates that local and global structures of ligands and motif sequences of GPCR are good features for prediction of strong binding.

## 2. Methods

### 2.1. Data Sources and Preprocessing

We downloaded data for 3055 GPCRs, 276,324 ligands, and 811,601 GPCR-ligand bindings from the GLASS database [9]. Among these, we included in the analysis only 213,918 ligands with a molecular weight greater than 150 and less than 500, since ligands that are too light or too heavy are likely to be ions or proteins, respectively. Accordingly, the number of final GPCR-ligand bindings was reduced to 303,587.

To obtain classification features from GPCRs, we first measure 1-amino frequencies (total 20) and 2-amino frequencies (total 400) for each GPCR. We also count the motif frequency of each GPCR. The motifs are small amino acid sequences that are specific to GPCRs and not observed in non-GPCRs. We use the MEME Suite [10] to obtain these motifs. We then exclude motifs with frequencies in all GPCRs of 1, resulting in a number of final unique motifs of 1,929. After counting the frequencies of 1,929 motifs, we have a vector with a length of 2,529 (=20 + 400 + 1,929), for each GPCR.

To obtain features from ligands, we first use four measurements: molecular weight, XlogP (a measure of the molecule's lipophilicity and solubility), hydrogen bond donors, and hydrogen bond acceptors. We name these four measurements as* 4chars*. We also use a canonical simplified molecular-input line-entry system (SMILES) of a ligand, which can be parsed into a graph of which nodes and edges represent atoms and bonding, respectively.

**Figure 1:**
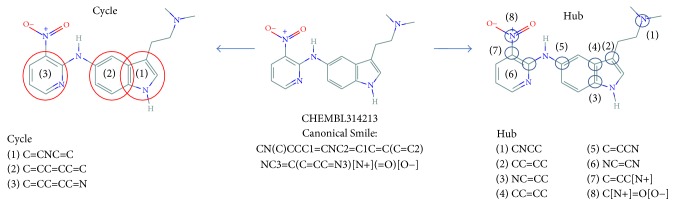
Examples of hub and cycles and how they are encoded.

From a graph of a ligand, we obtain two important features: a hub and a cycle. We define a hub as a node that has connection to more than 3 nodes. A hub structure is encoded as “*hub_node*–*A*=*B*≡*C*…,” where* hub_node* is a hub node, *A* is the satellite node first in alphabetical order, “–” is one of the bonding types between a hub node and* A*,* B* is located next to *A* in clockwise order, “=” is one of the bonding types between a hub node and* B*,* C* is located next to *B* in clockwise order, and so on.  Figure 1 shows 8 hub structures in the ligand (CHEMBL314213) and how they are encoded.

Cycles are mostly various kinds of benzene rings. Depth-first search (DFS) is used to search cycles from a graph. A cycle is encoded as “*A*–*B*=*C* …,” where *A* is the node first in alphabetical order, “–” is one of the bonding types between *A* and* B*,* B* is located next to *A* in clockwise order, “=” is one of the bonding types between *B* and* C*, and so on.  Figure 1 shows 3 cycles in CHEMBL314213 and how they are encoded.

We were able to obtain 1,031 unique hub structures and 1,741 unique cycle structures from 213,919 ligands. We then count the frequencies of those hubs and cycles for each ligand, so each ligand has a vector with a length of 2,776 (=1,031 + 1,741 + 4).

Each GPCR-ligand binding has two vectors, one from a GPCR and the other from a ligand, and we concatenate those two vectors. Therefore, each GPCR-ligand binding now has one vector with a length of 5,306 (=2,529 + 2,776).

### 2.2. Training Procedure

To obtain training data, we first randomly select 10,000 GPCR-ligand bindings and label them as positive. However, to train a classification model that distinguishes binding and nonbinding, we also need negative samples, which means nonbinding GPCR-ligand pairs. We used Algorithm 1 to generate 10,000 negative samples.

## 3. Results

We created 10 datasets, each of which consists of 10,000 positive and 10,000 negative samples, using all the attributes, all GPCR features and cycle, hub, and* 4chars* for ligand features. To select a classification algorithm, we compared 10-fold cross-validation results using various algorithms including SVM [11], naïve Bayesian [12], classification and regression tree (CART) decision tree [13], random forest [14], and neural network [15], all of which are implemented in scikit-learn [16]. We can see that random forest shows the best classification accuracy.

**Figure 2:**
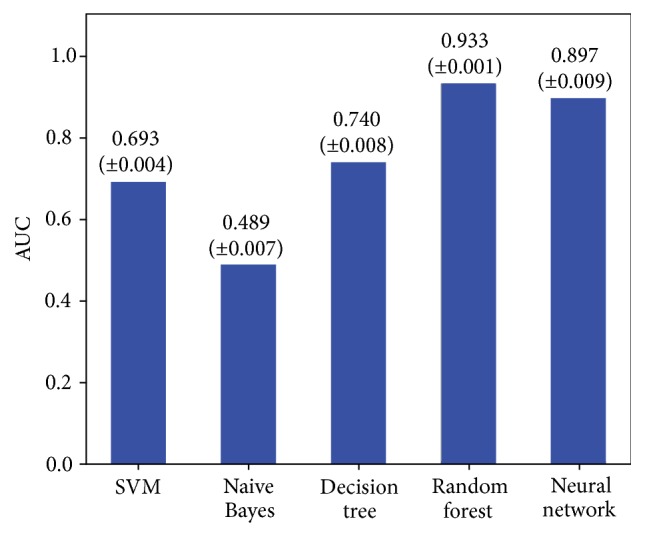
Classification results for various algorithms.

**Figure 3:**
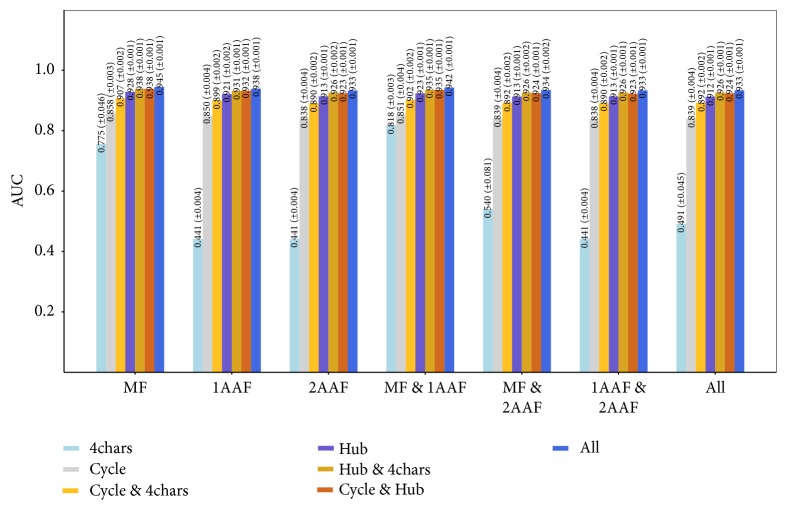
Classification results for all combinations of feature sets.

Next we performed 10-fold cross-validation for 10 datasets using all combinations of feature sets, as shown in Table 1. We used random forest, as it shows the best performance in Figure 2.  Figure 3 shows the classification results.

Figure 3 shows that using all ligand feature sets results in the highest accuracy for all cases. Among ligand features, hub seems to be most important, since “Hub,” “Hub &* 4chars*,” and “Cycle & Hub” are ranked 4, 3, and 2, respectively. Using “Cycle” was also relatively effective compared to “*4char*” which showed worst classification accuracy. These results show that structural data regarding ligands are key features.

Additionally, we can observe that the classification results using GPCR motifs only (first group) were the most accurate. This finding implies that motif sequences of GPCRs may play a role in ligand binding, and motif information can hold global structural information of GPCRs. We suspect that the amino acid frequency information may mask the motif information, since the classification results with GPCR motifs only show higher AUC than other cases. We can see that “MF & 1AAF” shows next best performance and “1AAF” shows better performance than “2AFF.” Those results imply that 1AAF is more important than 2AAF. When only “*4chars*” are used, “MF & 1AAF” show much higher AUC than other cases, which means 1AAF can be supportive when ligand information is not enough. From those results, we can conclude that GPCR motifs are more efficient features than amino acid frequencies in prediction of binding.

**Figure 4:**
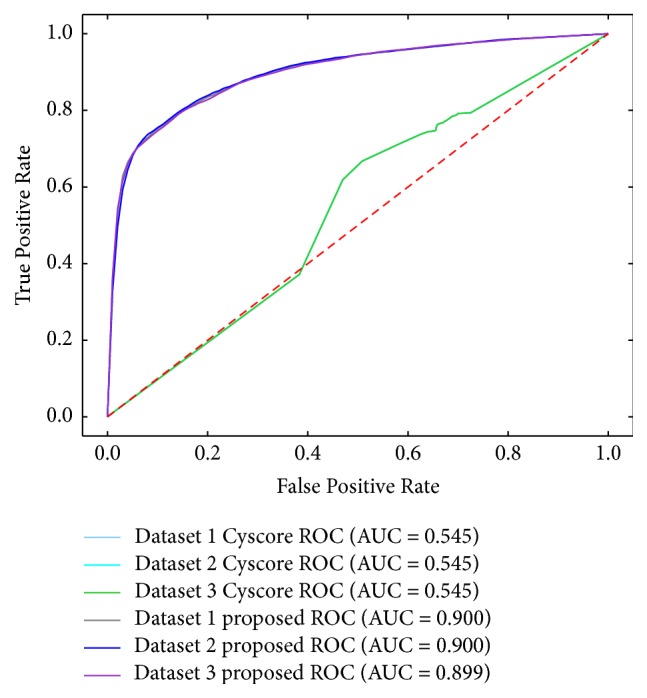
Comparison of prediction performance.

We compared the prediction performance of the proposed approach with Cyscore [1]. Since Cyscore requires 3D structure of the receptors, we generated GPCR with known 3D structure from PDB (http://www.rcsb.org) [17] and their interacting ligand pairs of which number is 110,186. The same number of negative samples was also generated according to Algorithm 1. Then we randomly selected three training datasets which is composed of 10,000 positive and 10,000 negative samples. The results of Cyscore are affinity scores of GPCR-ligand pairs, so we can figure out TPR (True Positive Rate) and FPR (False Positive Rate) values varying the affinity thresholds, draw ROC curve, and calculate AUC.  Figure 4 shows that our AUC values of the proposed approach are much better than those of Cyscore.

**Figure 5:**
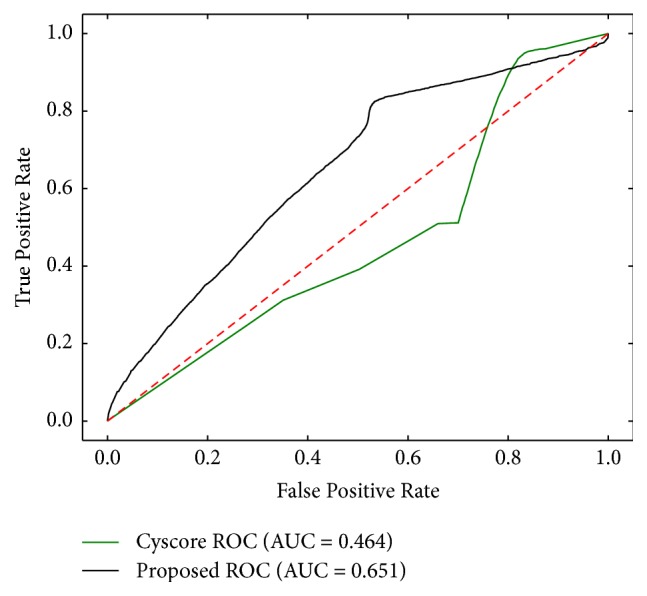
ROC curves for the predicted bindings.

Lastly, we randomly selected 100 GPCRs that do not have binding ligands provided in the GLASS database and predicted ligands that may bind to them (provided in the Supplementary Materials (available here)). To measure the prediction accuracy, we downloaded protein-ligand bindings from BindingDB [18]. We selected 9 GPCRs which are common in BindingDB, Cyscore, and the proposed bindings and counted the number of predictions found in the BindingDB. To count true negatives (which is not predicted by methods and also not in BindingDB), we define them as *X*  −  (true positives + false positives + false negatives), where *X* is a set of all possible bindings of 9 GPCRs and ligands which are bound by more than one of 9 GPCRs in BindingDB.  Figure 5 shows ROC curves for the predicted bindings of the proposed method and Cyscore, and we can see that AUC of the proposed method is much higher than that of Cyscore.

Among our predictions which were not found in BindingDB, we were able to identify some interesting bindings, one of which is “psychosine receptor (Q8IYL9), quercetin.” Studies have suggested that quercetin may have therapeutic benefit in cardiovascular diseases [19]. The psychosine receptor is a receptor for the glycosphingolipid psychosine (PSY) and several related glycosphingolipids. Interestingly, we were able to determine that GPR65 is associated with cardiovascular diseases [20]. Our prediction that quercetin binds to the psychosine receptor may assist in elucidating the role of GPCR65 in the cardiovascular diseases. Another interesting binding is “Frizzled-2 (Q14332), garcinone E.” Frizzled-2 is a receptor for Wnt proteins and has been reported to be elevated in metastatic liver, lung, colon, and breast cancer cell lines [21]. We found that garcinone E has already been proposed as a potential drug to treat certain types of cancer [22]. Our prediction that garcinone E binds to Frizzled-2 supports the rationale behind this suggestion and further suggests the hypothesis that Frizzled-2 may be inhibited by binding of garcinone E and may thus have a therapeutic effect in these types of cancer cells.

## 4. Discussion

In this paper, we propose a novel protein-ligand binding prediction method. The first step in this method is the extraction of local and global features from ligands and GPCR protein sequences. These features include hub and cycle structures of ligands and amino acid motifs of GPCRs. Our experimental results show that these new features were effective in predicting GPCR-ligand binding, and these features have the potential to enable us to discover hidden properties of good ligand-receptor bindings. Our method showed much higher AUC than Cyscore, and the suspected reason of low AUC of Cyscore is that it is not optimized for prediction of GPCR-ligand binding. Using the proposed method, we were able to identify novel ligand-GPCR bindings, some of which are supported by several previous studies.

## Figures and Tables

**Algorithm 1:**
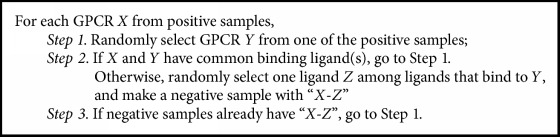
Algorithm for generating negative samples.

**Table 1:** Feature sets.

GPCR feature sets	Ligand feature sets
MF (motif frequency)	*4chars*
1AAF (1 amino acid frequency)	Hub
2AAF (2 amino acid frequency)	Cycle
MF & 1AAF	*4chars* & Hub
MF & 2AAF	*4chars* & Cycle
1AAF & 2AAF	Hub & Cycle
All	All

## Supplementary Materials

Supplementary Materials Each supplementary material file includes the ligands which are predicted to be bound by each GPCR. They are tab delimited files where first and second columns are chemical name and InChI Key, respectively (first row has the total number of predicted ligands).
